# Supplementary material for: Inhibition of overactive TGF-β attenuates progression of heterotopic ossification in mice
Source: Nat Commun. 2018 Feb 7;9:551. doi: 10.1038/s41467-018-02988-5 (PMC5803194; doi:10.1038/s41467-018-02988-5)
Supplement: Supplementary file 1 — Supplementary Information [file 41467_2018_2988_MOESM1_ESM.pdf]

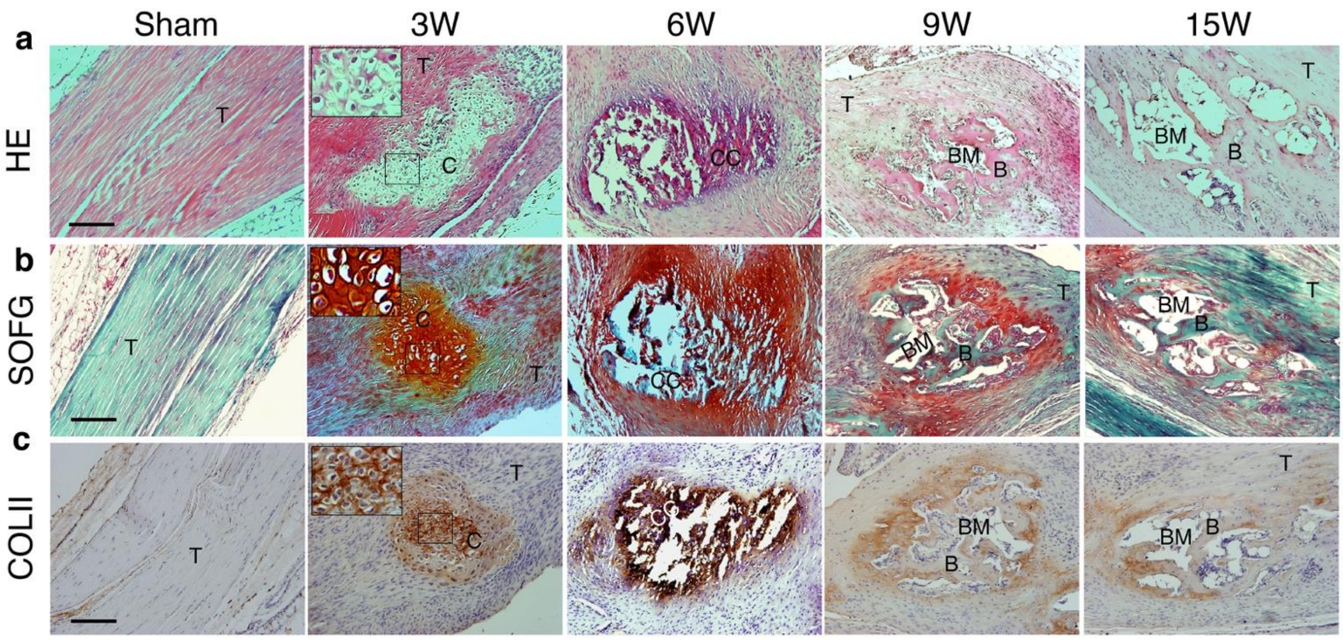

Supplementary Figure 1. Analysis of HO in Achilles tendon in ATP mice.

(**a**) H&E, (**b**) SOFG staining and (**c**) COLII immunostaining of Achilles tendon. Scale bars, 100μm. Magnified view of the boxed region of Achilles tendons 3 weeks after ATP were shown on the top left in the same figure. T, tendon; C, cartilage; CC, calcified cartilage; B, bone; BM, bone marrow.

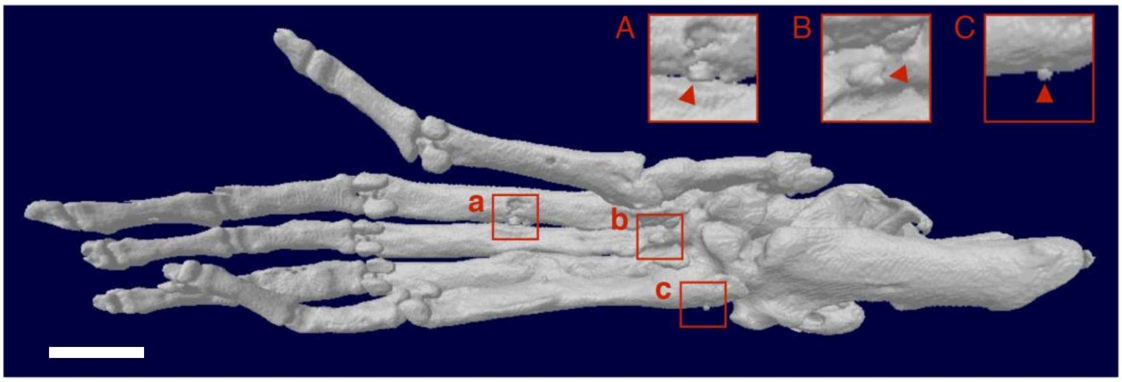

Supplementary Figure 2. Spontaneous HO in the ligaments of paws in 4-month-old CED mice.

(**a-c**) Ossified lesions were detected in ligaments of paws in 4-month-old CED mice. A-C are magnified view of the boxed region of HO lesions. Scar bar, 2mm.

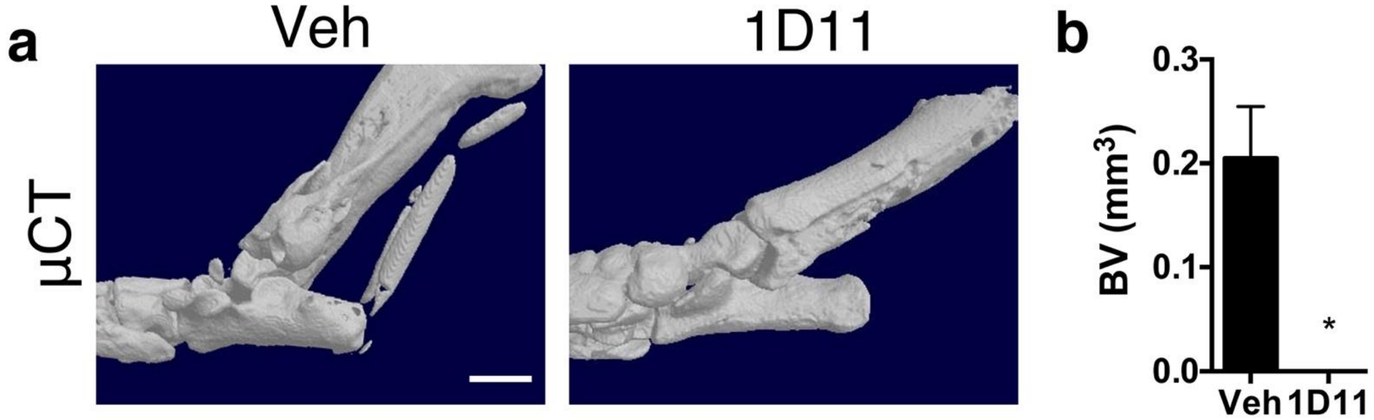

Supplementary Figure 3. Administration of TGF-β neutralizing antibody mitigates spontaneous HO in CED mice.

(**a**) Micro CT images of the Achilles tendon (sagittal view) of CED mice treated with vehicle or 1D11 daily for 4 weeks. (**b**) Quantitative analysis of bone volume of HO in Achilles tendon. Scale bar, 2mm. All data are shown as the mean ± s.d. n = 8 per group. *p<0.05 determined by two tailed, unpaired student's *t* test.

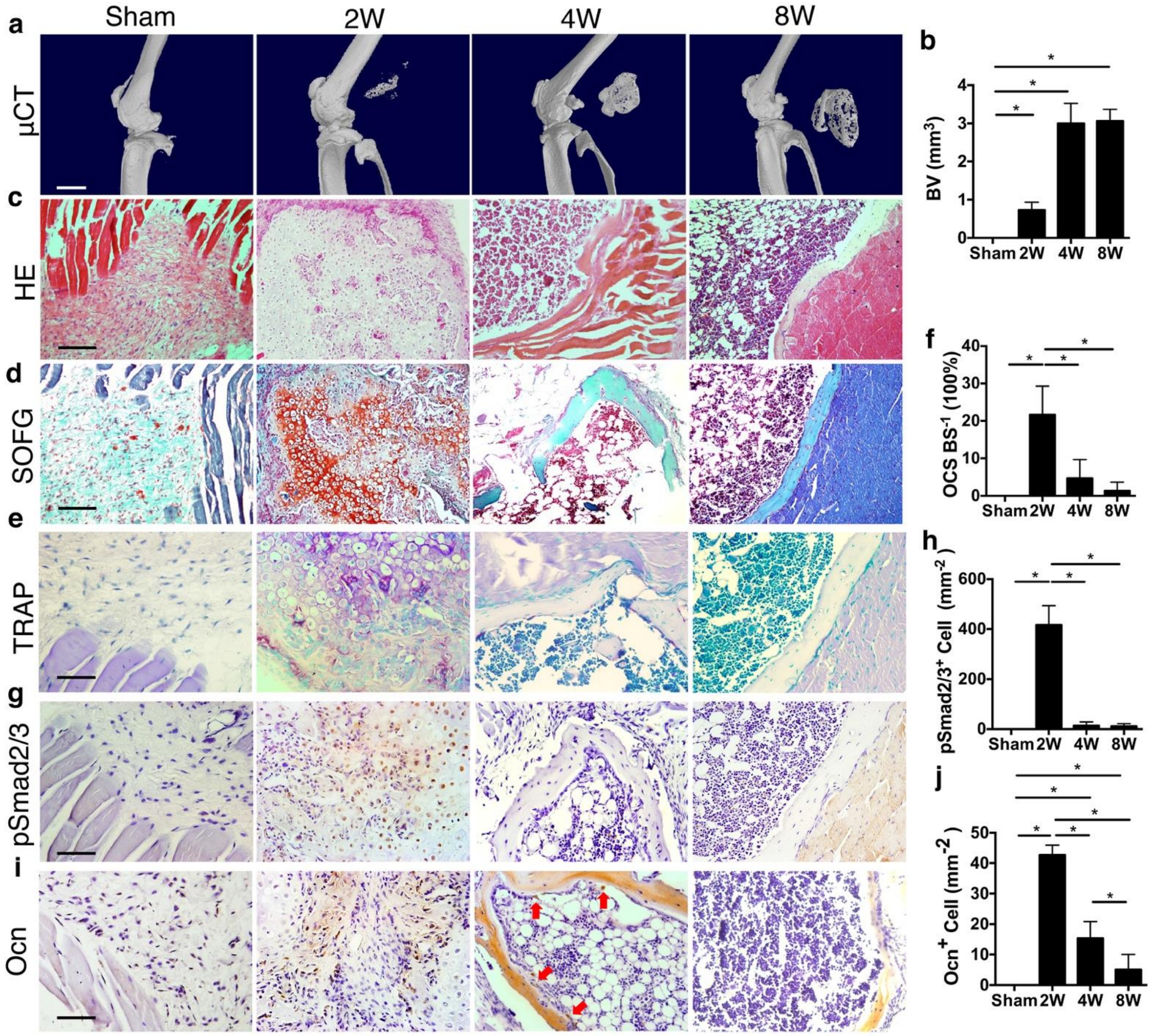

Supplementary Figure 4. TGF-β activity is increased during HO progression in BMP/Gelatin implantation.

(**a**) Micro CT images of hind limbs after sham operation or at 2, 4 and 8 weeks after BGI in hamstring muscles and (**b**) quantitative analysis of bone volume. Scale bar, 4mm. (**c**) H&E staining and (**d**) SOFG staining of hamstring muscles. Scale bars, 100µm. (**e**) TRAP staining (magenta) and (**f**) quantification of ectopic bone in mouse hamstring muscles. Scale bar, 50µm. (**g, i**) Immunohistochemical staining and (**h, j**) quantification of (**g, h**) pSmad2/3$^+$ cells and (**i, j**) Ocn$^+$ cells after sham operation or BGI. Scale bars, 50µm. Red arrow shows Ocn$^+$ cells. All data are shown as the mean ± s.d. n = 8 per group. *$p<0.05$ determined by one way ANOVA.

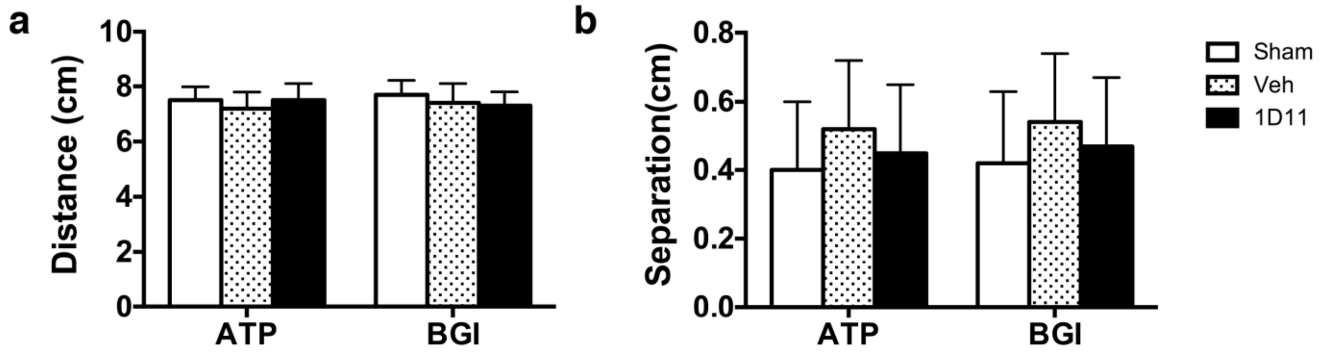

Supplementary Figure 5. Quantitative analysis of the walking footprint patterns of sham operated, vehicle or antibody treated ATP or BGI operated mice.

(**a**) The distance of stride length and (**b**) the distance between front and hind footprint were measured. All data are shown as the mean ± s.d. n = 8 per group. Statistical analysis was performed by one way ANOVA.

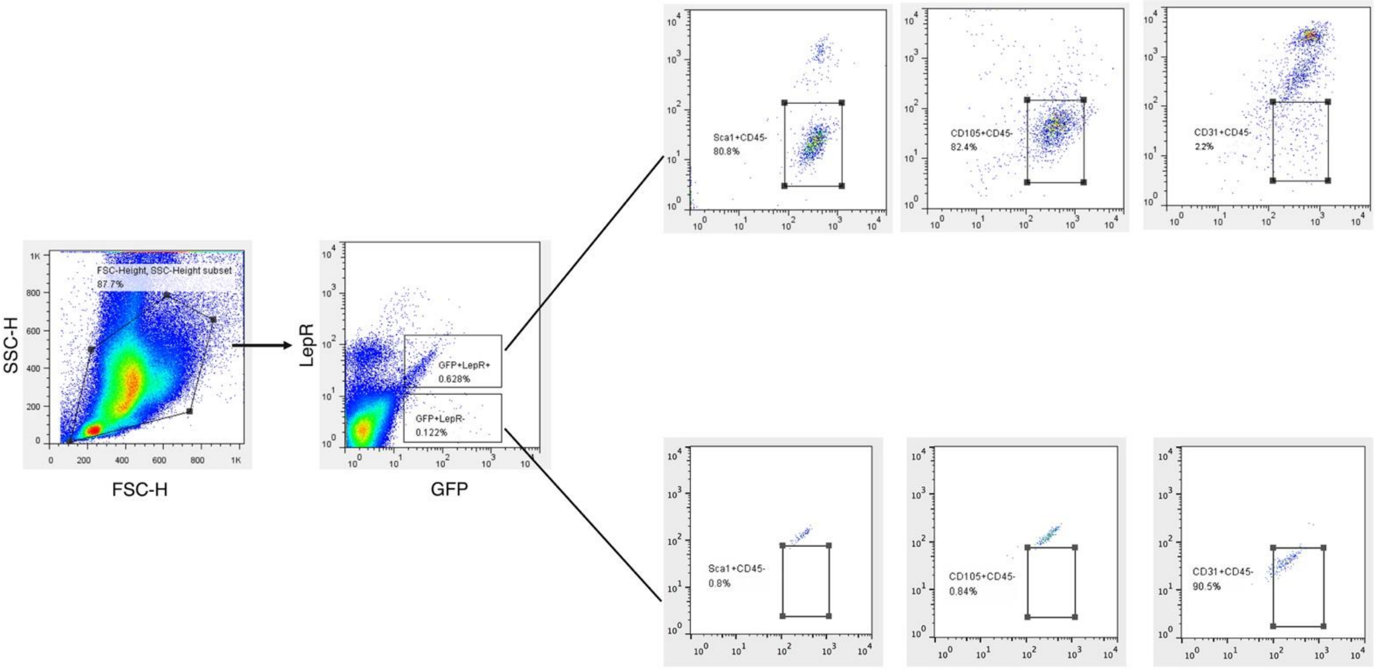

Supplementary Figure 6. Representative FACS gating strategy for analyzing Nestin^{+} cells.

Viable single cells were first gated to select GFP^{+}LepR^{+} and GFP^{+}LepR^{-} cells, and then CD45^{-}Sca1^{+}, CD45^{-}CD105^{+} or CD45^{-}CD31^{+} cells.

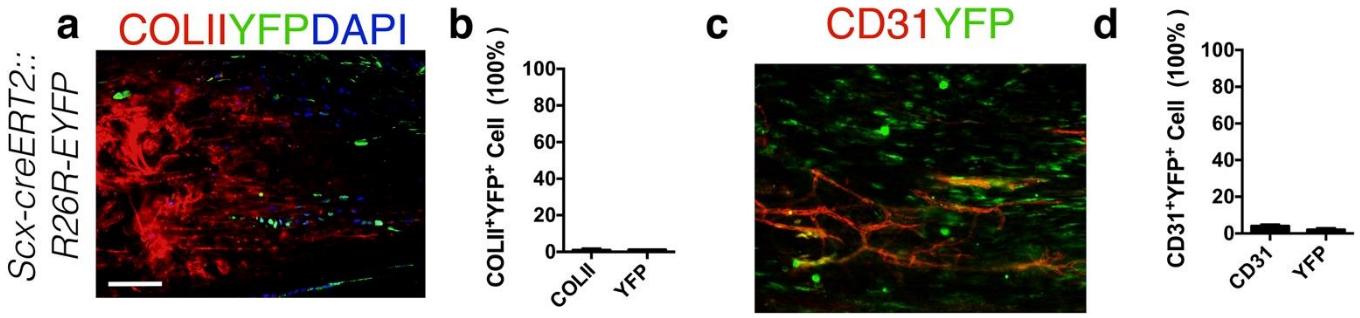

Supplementary Figure 7. Tendon residing Scx$^+$ cells do not provide precursors for HO formation.

(**a**) Immunostaining of COLII$^+$ cells (red) and YFP$^+$ cells (green) and (**b)** quantitation of *Scx-creERT2::R26R-EYFP* mice 3 weeks post ATP. Scale bar, 50μm, n = 8 per group. (**c**) CD31$^+$ cells (red) and YFP$^+$ cells (green) and (**d**) quantitation in ectopic bone marrow of *Scx-creERT2::R26R-EYFP* mice 6 weeks post ATP. Scale bar, 50μm, n = 8 per group. All data are shown as the mean ± s.d. n = 8 per group. Statistical analysis was performed by two tailed, unpaired student's *t* test.

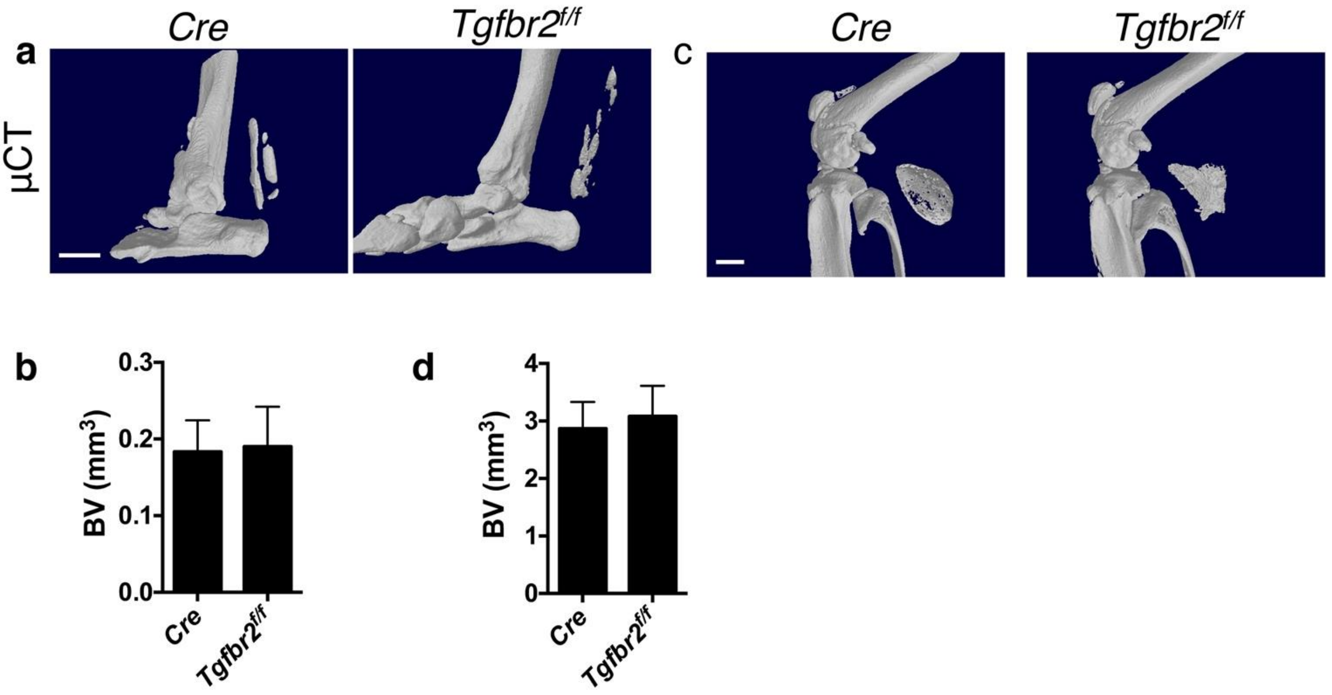

Supplementary Figure 8. No significant differences between tamoxifen treated *Nestin-creERT2* mice (*Cre*) mice and vehicle treated *Nestin-creERT2*::*Tgfbr2*$^{f/f}$ (*Tgfbr2*$^{f/f}$) mice.

(**a, c**) Micro CT images and (**b, d**) quantifications of (**a, b**) Achilles tendons or (**c, d**) hamstring muscles of tamoxifen treated *Nestin-creERT2* mice (*Cre*) mice and vehicle treated *Nestin-creERT2::Tgfbr2*$^{flox/flox}$ (*Tgfbr2*$^{f/f}$) mice for 2 months after undergoing ATP or BGI surgery, respectively. Scale bars, 2mm. All data are shown as the mean ± s.d. n = 8 per group. Statistical analysis was performed by two tailed, unpaired student's *t* test.
